# Implementation of patient-reported outcome measures in oncology practice: a communication-centered qualitative study on patient and healthcare professional perspectives

**DOI:** 10.1007/s11136-026-04378-7

**Published:** 2026-08-22

**Authors:** Linwei He, Anouk E. W. Teunissen, Nadine Bol, Kelly M. de Ligt, Emiel Krahmer

**Affiliations:** 1 Department of Communication and Cognition, Tilburg School of Humanities and Digital Sciences, Tilburg University, Tilburg, The Netherlands; 2 Department of Psychosocial Research and Epidemiology, Netherlands Cancer Institute, Amsterdam, The Netherlands; 3 Department of Medical and Clinical Psychology, Tilburg University, Tilburg, The Netherlands

**Keywords:** Patient-reported outcome measures, Implementation process, Health communication, Oncology care, Quality of life

## Abstract

**Purpose:**

Patient-reported outcome measures (PROMs) are increasingly used in oncology to support patient-centered care, yet their implementation in routine practice remains inconsistent. Using a communication-centered perspective which conceptualizes PROMs implementation as a process of meaning exchange between patients, clinicians, and implementation staff, this study examined PROMs implementation as a multi-stage process and explored how it unfolds in real-life oncology care, drawing on both patient and healthcare professional perspectives.

**Methods:**

A qualitative interview study was conducted in the Netherlands with patients with cancer (*n* = 10) and healthcare professionals and implementation staff in oncology care (*n* = 7). Semi-structured interviews were informed by a communication-centered framework conceptualizing PROMs implementation as a multi-stage process. Data were analyzed using hybrid inductive-deductive thematic analysis.

**Results:**

Six interrelated stages of PROMs implementation were identified: (1) selecting and organizing PROMs use, (2) establishing purpose and motivation, (3) experiencing PROMs as a task, (4) making sense of results together, (5) applying PROMs information in care, and (6) evaluating and improving PROMs implementation. Findings showed that experiences at different stages were interconnected: processes occurring earlier in the implementation influence how PROMs were later understood, discussed, and used in care. Across roles, participants recognized PROMs’ potential to support communication beyond clinical indicators, while also describing frustration when PROMs were collected but not meaningfully used. Patient and clinician engagement influenced one another across stages, reflecting the reciprocal nature of PROMs as a communication tool.

**Conclusion:**

PROMs implementation should be viewed as an interdependent, sequential process. Improving connections between stages is essential to realizing PROMs’ communicative and patient-centered potential in oncology care. Practical strategies such as personalizing PROMs invitations, completing the feedback loop between PROMs results and patients, and integrating supportive tools for clinicians can help translate PROMs data into meaningful patient-centered dialogue.

**Supplementary Information:**

The online version contains supplementary material available at https://doi.org/10.1007/s11136-026-04378-7.

## Abstract (plain language summary)

Patient-reported outcome measures (PROMs)—questionnaires about symptoms and quality of life—are increasingly used in cancer care. These questionnaires, often completed by patients before or between appointments, are meant to help healthcare professionals understand how patients are really doing beyond medical test results. However, in daily practice, these questionnaires are not always used in a consistent or meaningful way. This study was needed to understand why this happens.

We examined how these questionnaires are introduced, completed, discussed, and used in real-life oncology care. In particular, we asked: at which points in this process do problems arise, and how do these problems affect patient care?

We interviewed cancer patients and healthcare professionals in the Netherlands. We found that using these questionnaires is not a single action, but a series of connected steps. When early steps, such as clearly explaining the purpose, are not well communicated, later steps, such as discussing results during consultations, are also less effective. Both patients and professionals acknowledged the potential of these questionnaires to improve communication, but they were frustrated when answers were collected without follow-up.

Our findings suggest that improving how these steps connect in practice can help turn questionnaires into more meaningful conversations and more patient-centered cancer care.

**Supplementary Information:**

The online version contains supplementary material available at https://doi.org/10.1007/s11136-026-04378-7.

## Introduction

Patient-reported outcome measures (PROMs) are instruments, often validated questionnaires, that capture a range of outcomes including symptoms, functioning, quality of life, psychological well-being, and treatment-related side effects [1, 2]. PROMs have become essential in cancer care because they offer insights into aspects of patients’ health that may not be observed through clinical examination alone [3]. Research in oncology and quality of life shows that the use of PROMs can improve symptom management [4], enhance patient-clinician communication [5], and support more responsive and person-centered care [6]. At the organizational level, PROMs data can be used to inform quality improvement and monitor outcomes across care processes [7, 8]. Beyond their measurement function, PROMs are also communicative tools that elicit patients’ perspectives and bring these perspectives into clinical encounters [9].

Despite these benefits, integrating PROMs meaningfully into routine oncology practice remains challenging. Implementation across hospitals has shown wide variation in completion rates, patient satisfaction, and the extent to which PROMs are discussed in clinical encounters [10–12]. Some studies report that patients do not always receive feedback about their responses, and that clinicians differ in whether they incorporate it into consultations or how they interpret the data [13, 14]. These inconsistencies indicate that, although PROMs bring added value to routine cancer care, their full potential is yet to be realized.

Earlier research has pointed to barriers such as system integration, inadequate digital infrastructure, and logistical challenges [15–17]. However, a solely barrier-oriented perspective might overlook how PROMs’ value depends on a sequence of interconnected communication processes, from inviting patients to complete PROMs and interpreting results, to discussing them during consultations and informing care decisions [18, 19]. In oncology, where care pathways are complex and patients often experience high symptom burdens, these processes are especially important. When they are fragmented, or insufficiently connected with one other, PROMs risk becoming a burden to patients and clinicians rather than supporting meaningful communication [20]. Therefore, this study asks: where do challenges in PROMs implementation emerge across the implementation process, and how do these challenges shape the communicative use of PROMs in oncology care?

Recent conceptual research has emphasized this communicative and procedural dimension. De Ligt and colleagues [19] proposed a framework that conceptualizes PROMs use as a multi-stage communication process involving the exchange of meaning between patients, clinicians, and support staff. The framework proposes several key stages, such as how the purpose of PROMs is introduced, how results are made accessible and understandable, and how they are brought into conversations about care. Importantly, these stages are sequential and interdependent. For instance, prior research suggests that the value of PROMs depends not only on their completion but on how results are fed back and used in clinical encounters [21, 22]. Without such feedback, response rates may decline over time, further limiting the data available for clinical use [12]. Together, these stages form a workflow for PROMs implementation, highlighting specific process points where PROMs can enable or hinder meaningful dialogue.

While this framework offers a valuable theoretical lens, it remains largely conceptual, and empirical examinations of how these communication processes unfold in real-life oncology care remain limited. In particular, we lack insight into which steps are involved in PROMs implementation, where challenges tend to emerge along this process, and why the anticipated communicative and patient-centered benefits are difficult to achieve. Drawing on the framework proposed by De Ligt et al. [19], this study aims to (1) use a communication-centered analytical lens to examine how PROMs are implemented across sequential stages in everyday oncology care, and (2) contribute to the framework’s refinement by providing empirical validation and identifying steps that extend beyond its current scope. While our perspective is communication-centered, it also encompasses systemic and organizational factors insofar as they impact how PROMs-related communication unfolds. Building on theoretical insights, this study aims to examine what it takes to use PROMs to facilitate meaningful communication between patients and healthcare professionals within the complexity of cancer care.

## Methods

### Study design

This qualitative exploratory study, situated within an interpretivist paradigm, was designed to generate in-depth understanding of a process that has received limited empirical attention. To this end, individual semi-structured interviews were conducted to examine how PROMs are currently experienced in oncology care in the Netherlands, which bottlenecks are encountered, and where in the implementation process they emerge. This study is reported according to the Standards for Reporting Qualitative Research [23]. Ethics approval was obtained from the Research Ethics and Data Management Committee of Tilburg Universtiy (approval code: REDC 2024.83) and the study was conducted in compliance with the ethical and data management regulations of the school. Written informed consent was obtained from participants prior to data collection.

### Interview guide

**Fig. 1:**
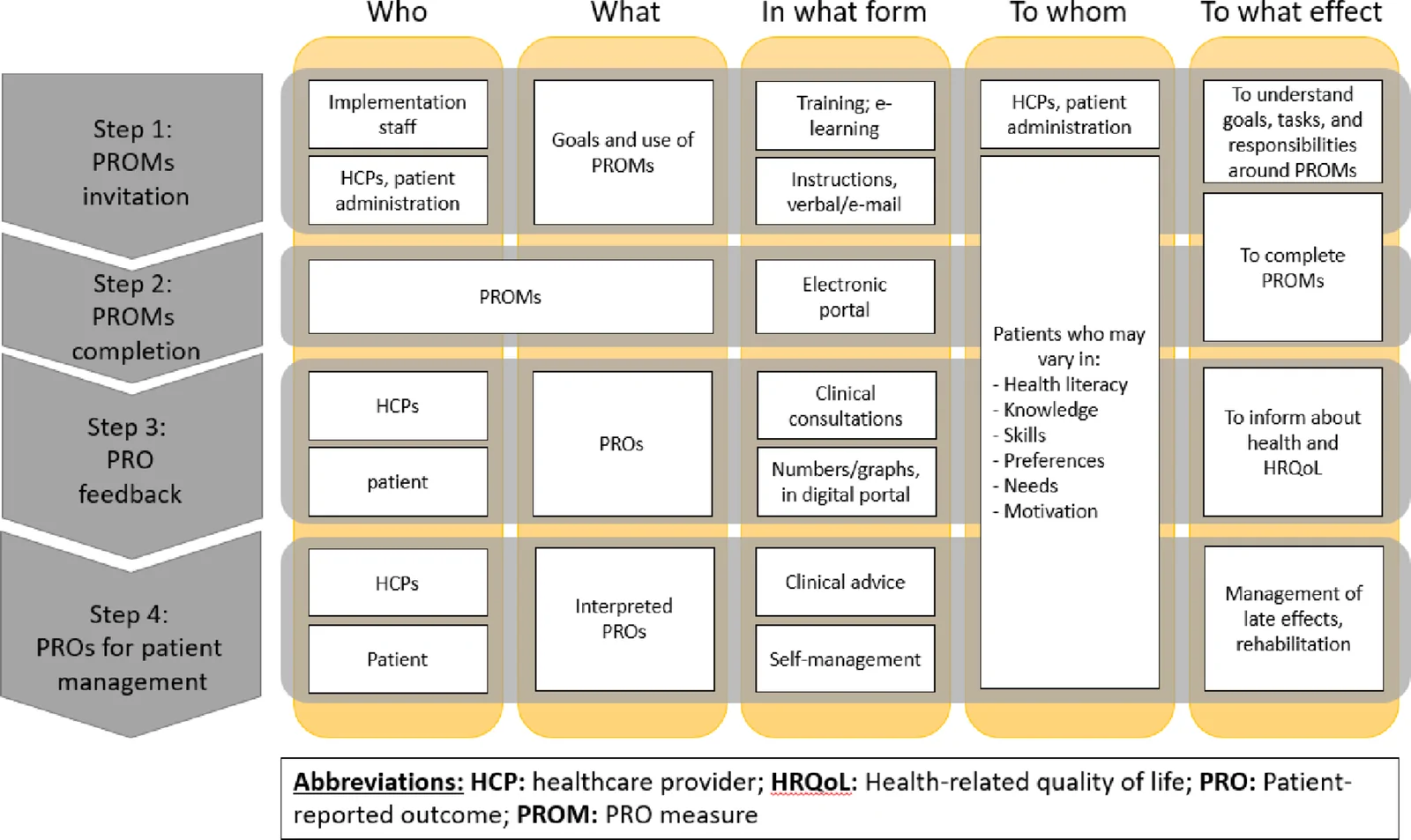
A communication-centered framework of PROMs implementation [19]. Reproduced from De Ligt et al., *J Med Internet Res* 2025; 27: e60777 (https://www.jmir.org/2025/1/e60777). © 2025 De Ligt et al. Licensed under CC BY 4.0

The semi-structured interviews followed a predefined interview guide, allowing the interviewers to follow core topics while remaining flexible to capture additional insights raised spontaneously by participants. The interview guide was based on the communication-centered framework of De Ligt et al. [19], grounded in Lasswell’s classical communication model [24], which conceptualizes communication processes through the questions “who does what, to whom, in what form, and with what effect.” (See Fig. 1 for an overview of the framework^1^). Building on these principles, our interview guide asked participants to identify the steps they have experienced in PROMs-related activities, and reflect on *who* was involved, *what* actions were taken at different points in the process, *in what form* information was communicated, and *with what* intended and perceived *effects*. Participants were encouraged to discuss their experience related to PROMs, challenges encountered, and desired solutions or opportunities at each step of the process. Prior to data collection, two interviewers (LH and AT) conducted practice sessions to test the clarity of the interview guide to ensure that it elicited the type and depth of information needed for the study. The interview guide is provided in Online Resource 1.

### Participants

Participants included three groups: (1) clinicians working in oncology departments of hospitals and cancer centers in the Netherlands; (2) PROMs implementation staffs, such as policy advisors and coordinators; and (3) cancer patients or survivors. Clinicians and staffs were recruited through the research team’s professional networks using purposive and convenience sampling to ensure variation in institutional type, oncology specialties, and roles in PROMs implementation. Patients were recruited through a Dutch online platform (Kanker.nl) for individuals with lived experience of cancer.

A total of 7 professionals and 10 patients participated in the interview. Professional participants represented a range of oncology specialties, including head and neck oncology (*n* = 2), gynae-oncology (*n* = 1), neuro-oncology (*n* = 3), and gastrointestinal oncology (*n* = 2)^2^, working across four different organizations in the Netherlands: two general hospitals, one comprehensive cancer center, and one specialized radiotherapy institute. This group also included experts involved in PROMs implementation (*n* = 1) and policy advisory work (*n* = 1). Years of clinical or implementation experience ranged from 4 to 20 years. In these settings, PROMs were typically administered automatically through electronic health record (EHR) portals or separate online platforms. Clinicians used PROMs results during consultations when accessible; the implementation specialist coordinated PROMs use and dashboard management, and the policy advisor coordinated instrument selection and consulted with national quality registries.

The patient sample included individuals at different stages of cancer journey: some were in active treatment (*n* = 6), others had completed treatment (*n* = 3), and one participant did not specify their treatment status. Participants represented diverse cancer types, including breast cancer (*n* = 6), prostate cancer (*n* = 1), rectal cancer (*n* = 1), and thyroid cancer (*n* = 2). They were treated in at least seven different institutions across six provinces, with no systematic overlap with professional participants’ settings. Time since diagnosis ranged from 4 months to 10 years. Patients varied in their prior experience with PROMs. Most had experience with quality-of-life or symptom questionnaires on multiple occasions as part of routine care, while two patients recalled only pre-treatment screening forms or general health questionnaires. When patients did not spontaneously distinguish PROMs from other questionnaires, we probed them to reflect on these specifically.

Sample size was guided by the concept of information power, a theory for determining sample size in qualitative research that emphasizes the specificity and diversity of information rather than numerical saturation [25]. In this study, information power was supported by the combination of a focused study aim, a guiding theoretical framework that structured the inquiry, the inclusion of participants with complementary perspectives on the same implementation process (patients, clinicians, and implementation/policy professionals), and the specificity of the PROMs implementation context in oncology care.

### Data collection

Interviews were conducted between January 2025 and July 2025 by two interviewers (LH and AT). Interviews with professionals were conducted in English, interviews with patients in Dutch. The interviews lasted 30 to 60 min each and were conducted online via Microsoft Teams.

Interviews were audio-recorded and transcribed. Dutch transcripts were translated into English for analysis. Participants were offered the opportunity to receive a copy of their interview transcript to check for accuracy. One participant reviewed and confirmed the accuracy of the transcript; no changes were requested.

### Data analysis

Transcribed data were analyzed using reflexive thematic analysis [26, 27]. We adopted a hybrid approach combining deductive and inductive coding [28]. The deductive component entailed that when coding, we used the framework’s stages [19] as coding categories where applicable. For example, content about how purpose was communicated or whether results were discussed was coded in relation to the corresponding stage. This did not involve a predetermined codebook. Alongside this, inductive codes emerged freely for content that did not map onto the framework. Themes were then developed inductively by grouping codes, and subsequently mapped (but not forced) onto the framework to examine where they aligned with, or extended beyond, the sequential stages.

Two researchers independently coded an initial subset of transcript and compared their coding to align on the analytical approach. Subsequent coding was then performed in an iterative process over multiple reads of the transcripts. Coding and data management were performed using ATLAS.ti (version 26) [29]. The first author developed a preliminary thematic structure by grouping codes with shared meanings, which was discussed and refined with all co-authors across multiple rounds. In line with the principles of reflexive thematic analysis [23], we acknowledge the researchers’ positioning in shaping the analysis. The interviewers (LH and AT) have backgrounds in health communication and PROMs communication in oncology, which informed their sensitivity to communicative processes but may also have oriented attention toward communication-related challenges. The author team includes expertise in PROMs implementation in clinical practice (KdL), whose perspectives helped balance the analytical lens during the collaborative discussions in which themes were refined.

## Results

### Overview of themes

**Fig. 2:**
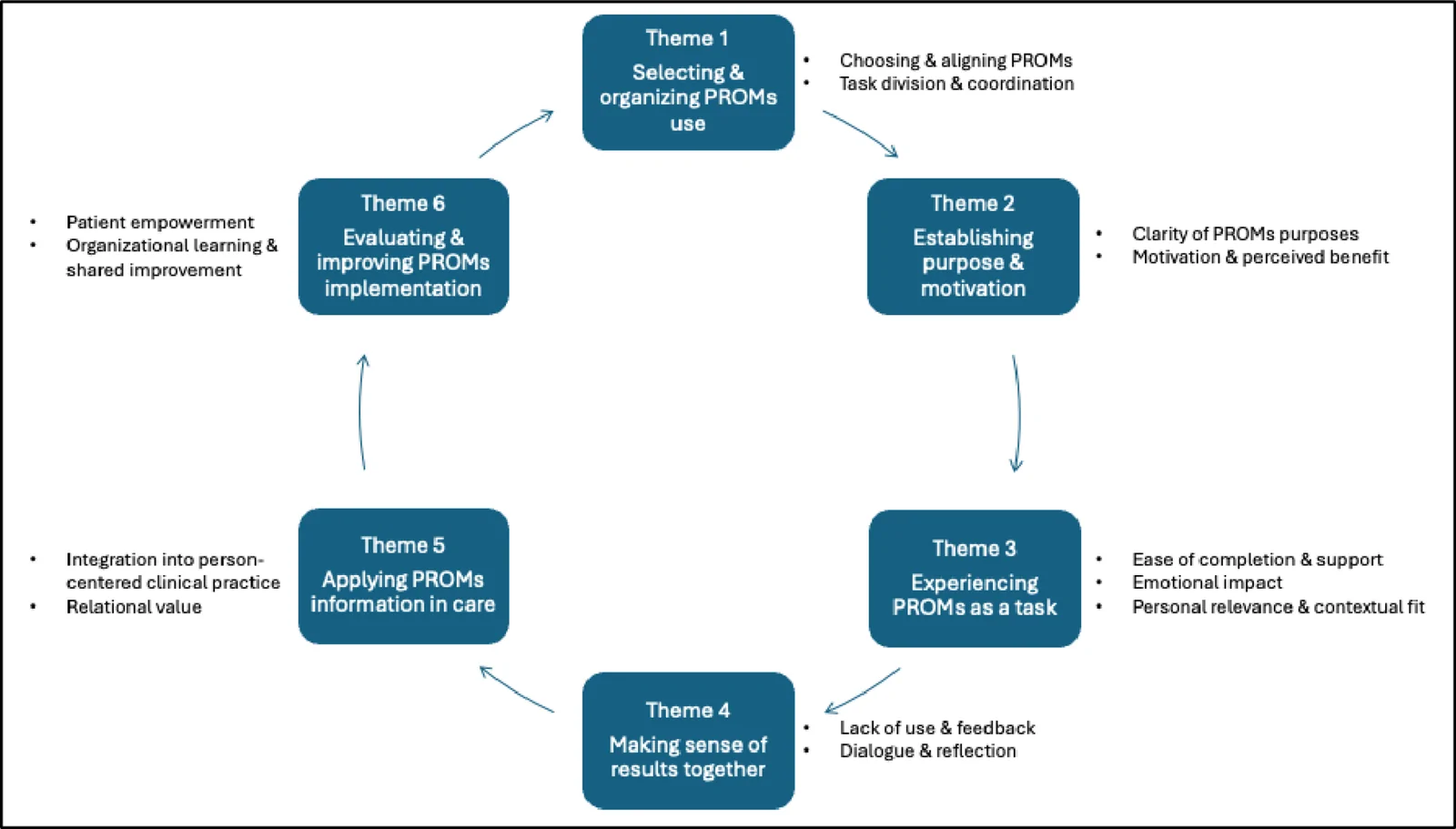
Illustration of the sequential process of PROMs implementation formed by the themes

The inductive-deductive thematic analysis resulted in six themes, each representing a stage in PROMs implementation (see Fig. 2 for a visual overview). While the interview guide was inspired by the sequential, communication-oriented framework [19], participants expanded, refined, and re-specified these stages based on their experiences, resulting in an empirical elaboration of the original framework depicted in Fig. 1. Below, the six themes are discussed, with detailed results per theme in Table 1.

### Theme 1: selecting and organizing PROMs use

The first theme captures a preparatory phase that precedes patient involvement. This theme was only mentioned by the professional participants, while patients generally were not aware of this stage. Professionals described this as foundational for making the following steps possible. They highlighted that selecting appropriate PROMs instruments and embedding them into clear clinical workflows requires efforts at the organizational level, yet this process is often fragmented and inconsistent.

Professionals describe the selection of PROMs (subtheme 1.1) as a decision shaped by local preference, previous research, or regional and national discussions. This led to significant variation between institutions and disease areas. For clinicians, this variation and lack of consensus created confusion about which questionnaires should be used and when, as well as extra burden to find agreement. A subtle difference regrading PROMs selection emerged across professional roles: the policy advisor described a deliberate process of consulting validated instruments and national data dictionaries: “*We do a search with our hospital-wide quality department on what kind of questionnaires are used for this tumor group*” (P6), while clinicians generally described PROMs as given tools: “*There are three questionnaires we can use or we have to use*” (P1), without much engagement in the selection process.

Task division (subtheme 1.2) emerged as another challenge. PROMs-related responsibilities, such as sending invitation, monitoring completions, and checking alerts, were distributed across nurses, data specialists, and clinicians. However, role division is different between institutions, and many patients move across hospitals during their treatment. These institutional differences are not always aligned, which complicated communication and coordination. As one clinician illustrated, “*In oncology*,* about 60% patients are surgically treated in a different hospital and then come back (to us) for follow up. The care processes are really*,* really complicated.”* (P2).

**Table 1:** Overview of themes, subthemes, and example quotes

Theme	Subtheme	Example quote
1. Selecting & organizing PROMs use	1.1 Choosing & aligning PROMs	“*We do struggle sometimes … the number of questions we ask*,* whether we have to use validated questionnaire*,* or whether we should only focus on the data we think we need … it’s a constant discussion*” (P6, policy advisor)
	1.2 Task division & coordination	“*If they’ve had all their treatment with curative intent in Eindhoven (and not with us)*,* you have no clue how they’re doing*,* what type of person they are*,* and that’s really important information when you start your second line treatment*.” (P2, clinician)
2. Establishing purpose & motivation	2.1 Clarity of PROMs purpose	“*We’re a little pushed because of the quality indicator. We have to arrange it (PROMs).*” (P1, clinician) “*And only afterward I thought*,* why did I fill all that in*.” (P9, patient) *“(They are) not really for yourself*,* more that the healthcare provider can use them*.” (P17, patient)
	2.2 Motivation & perceived benefit	“*It becomes an administrative burden*,* because it comes with an extra consultation between the nurse with the patients*.” (P2, clinician) “*It’s always a balance between what’s extra work and what’s extra gain.”* (P5, clinician) “*I do think they (PROMs) are important to complete. With the other ones (questionnaires) I fill out*,* I always get feedback showing roughly where I stand.”* (P11, patient) “*I didn’t find it important (if no feedback is given)”* (P10, patient)
3. Experiencing PROMs as a task	3.1 Ease of completion & support	“*I think many patients can complete the questionnaires*,* but we have an exceptionally well-educated population*.” (P4, implementation staff) “*Most of the time they don’t have very high education or they don’t have education at all.”* (P1, clinician) “*The Dutch (language) used in the app is sometimes quite complex.”* (P9, patient) “*I’ve worked with people with intellectual disabilities … they don’t say ‘I don’t get it’*,* they come up with excuses. Then care providers might think they are not cooperating*,* but that’s not the case*.” (P9, patient)
	3.2 Emotional impact	“*Lately it’s also been mentally quite tough to go through all this stuff (questions) again and again.”* (P11, patient) “*I also have moments where I think ‘no*,* I’m not doing this (PROMs) now*,* it’s not going well now’*”. (P12, patient)
	3.3 Personal relevance & contextual fit	*“What I consider a livable life might be totally different for someone else.”* (P16, patient) “*If (questions) come from the prostate cancer foundation*,* those should be general questions to paint a general picture. (Those) from the hospital should be very specific to my life and my situation.”* (P13, patient)
4. Making sense of results together	4.1 Lack of use & feedback	“*We do know that we collect a lot of data which we don’t use.”* (P6, policy advisor) “*We don’t check if they (patients) fill it in. We’re not very pushy.”* (P1, clinician) “*I have no idea whether they see the results of those questionnaires. I haven’t received feedback on them.”* (P13, patient)
	4.2 Dialogue & reflection	“*I can see in the dashboard what is going well and not so well*,* and this is really helpful in opening the conversation.”* (P3, clinician) “*There are specific questions where I think ‘hey*,* maybe I can do something with that’.”* (P11, patient)
5. Applying PROMs information in care	5.1 Integration into person-centered clinical practice	“*It’s not so much integrated yet for the healthcare professionals*.” (P4, implementation staff) “*It takes a lot of work to navigate (the system) through the patient’s file to have the data you want.”* (P7, clinician) *“The other thing would be if you have a very red kind of answer*,* the ward is being warned*,* and people start calling the patient.”* (P5, clinician)
	5.2 Relational value	*“If someone lost her husband three months ago*,* that’s more important than the routine gynae care consultation.”* (P2, clinician) *“I think there is a personal contact with the nurse … she can ask*,* based on the questionnaire*,* ‘I see this*,* was it very unpleasant?’”* (P15, patient)
6. Evaluating & improving PROMs implementation	6.1 Patient empowerment	*“We could educate our patients more about what they could do to improve their health … they don’t recognize they have a role in this.”* (P4, implementation staff)
	6.2 Organizational learning & shared improvement	*“The purpose is to use these data for nationwide analysis … and see whether there are differences or where there can be improvement.”* (P7, clinician)

### Theme 2: establishing purpose and motivation

This theme captures the step of inviting patients to complete PROMs, corresponding to Step 1 in the theoretical framework as shown in Fig. 1. This is the first step in the data-collection process and a key moment deciding what information becomes available for care. Both professionals and patients emphasized that engaging with PROMs requires understanding *why* they are being completed and *for whom*, yet these purposes were not always clearly communicated or understood.

Professionals differed in how they described the purpose of PROMs (subtheme 2.1). Implementation and policy professionals articulated an institutional vision, including an explicit commitment to discussing PROMs results with patients and using data for quality improvement (P6). Clinicians also recognized PROMs’ clinical potential, but more often described the initial driver for adoption as external: “*We’re a little bit pushed because of the quality indicator*” (P1). PROMs invitations were often automated within digital systems, and clinicians did not always have the time or opportunity to introduce their purpose personally to patients, which made PROMs seem like administrative obligations.

Patients reported similar experiences regarding unclear purpose. They mentioned that the purpose of PROMs was rarely explained to them, and they spontaneously formed a range of own interpretations. These perceived purposes were diverse and ranged from research, quality improvement, to more clinical purposes such as medication monitoring, treatment guidance, and informing their physician. These interpretations often reflected what patients hoped PROMs would do, even if such uses did not always occur in practice.

Motivation to complete and use PROMs strongly depended on perceived benefits (subtheme 2.2). Patients mentioned that personal explanation from clinicians can increase willingness to engage, and PROMs felt worthwhile only when responses were followed by feedback or used in consultations. This anticipation of feedback as a motivational driver is distinct from the actual feedback practices described in Theme 4, though the two are closely connected: when feedback is absent in practice, it feeds back into patients’ motivation, reducing their willingness to complete PROMs in the first place. Altruism - wanting to use own data to help future patients - also encouraged completion. However, when PROMs did not lead to follow-up, patients questioned their relevance and became less engaged. As one patient put it, *“If something is really done with it*,* of course it’s important. But like I said… I never believe anything is actually done with them.”* (P14) Clinicians’ motivation was impacted by the tension between perceived benefit and experienced burden. When the added value of PROMs comes with extra workload, motivation is difficult to sustain: “*It’s always a balance of what’s extra work and what’s extra gain*” (P5).

### Theme 3: experiencing PROMs as a task

The third theme reflects the practical and emotional experience of completing PROMs, corresponding to Step 2 in the theoretical framework. While professionals recognized that PROMs should be accessible and easy to complete, patients provided detailed experiences of usability challenges, emotional burden, and mismatches between standard questionnaires and the realities of individual trajectory with cancer.

Professionals identified multiple factors regarding ease of completion and support (subtheme 3.1), such as digital literacy and language proficiency. They also described systemic barriers, such as fragmented digital platforms and insufficient integration with electronic health record systems. These structural issues often created additional work for both clinicians and patients. Patients similarly reported difficulties, including complex interfaces, multiple logins, and long questionnaires, which made completion burdensome at times.

Besides these practical barriers, patients also experience PROMs on an emotional level (subtheme 3.2). PROMs sometimes elicit reflections and a sense of being cared for, but also risk adding burden and fatigue, particularly when detailed symptom questions were asked repeatedly during periods of distress. As one patient noted, *“Because I am at the very end of the disease*,* questions about death are quite heavy.”* (P13).

Another factor emphasized by patients is the personal relevance and contextual fit of the questions (subtheme 3.3). Some standard questions did not apply to their situations, and this caused extra burden and hindered engagement. This observation was also echoed by clinicians, such that PROM questions should be adapted to individual patient situation: *“If I’m saying in my PROMs that I have troubles climbing the stairs*,* don’t ask me whether I can ride my bike.”* (P2).

### Theme 4: making sense of results together

The next stage concerns how PROMs results are interpreted and shared, related to but expanding Step 3 in the theoretical framework. Across interviews, both groups described a weak or inconsistent feedback loop (subtheme 4.1), where results were collected but not always discussed. Clinicians acknowledged that they did not always act upon PROMs data, often due to limited time and prioritizing other clinical results. Patients reported similar experiences from their side: many said that they rarely received feedback and were unsure whether clinicians had seen or used their responses, which made them question the value of completing PROMs. As one patient remarked, “*Then I arrived at the appointment*,* and the doctor hadn’t read them*,* or hadn’t even received them. Then I think ‘garbage in*,* garbage out’*,* right? If no one reads it*,* there’s not much point.*” (P11).

Professionals noted several reasons for such lack of feedback. Clinicians often attributed such inconsistent follow-up to inadequate tool support. PROMs results were usually presented as raw individual data, leaving clinicians having to interpret the data themselves, something they had little time for during brief consultations. As a result, they tended to focus on clinical indicators that were more meaningful. This lack of supportive tooling was linked to limited ICT involvement, such as the absence of automated analysis or graphical summaries, which clinicians associated with low institutional prioritization of PROMs. *“It would be the ideal situation that those tables would help us in selecting what patient needs or not … but we’re not using it yet due to our lack of innovation power. ”* (P5).

Despite these challenges, both patients and professionals acknowledged the importance of providing feedback (subtheme 4.2). Patients described feedback as an encouragement for self-reflection and a source of relational care, such that they felt “seen” when clinicians discussed their PROMs responses. Healthcare professionals recognized that discussing PROMs results improves patient response rates and enriches conversations by bringing up topics that might be overlooked by only clinical examinations.

### Theme 5: applying PROMs information in care

This theme reflects the translation of PROMs information into clinical practice, related to Step 4 in the theoretical framework. Clinicians described PROMs as potentially useful for triage, monitoring symptoms, support self-management, and initiating referrals (subtheme 5.1). However, this integration varied widely. In ideal cases, PROMs prompt clinicians to address issues beyond clinical indicators, creating opportunities for tailored care. But more commonly, professionals reported that PROMs data ran parallel to clinical workflows rather than being embedded within them. As one implementation staff observed, “*healthcare professionals are a bit reluctant to use the PROMs information during consultations with patients.*” (P4). Patients often expressed desires for actionable follow-up based on their PROMs results: personalized suggestions, credible information source, or self-management strategies are valued outside of the consultation room.

Besides the clinical usefulness, both groups emphasized the importance of human contact beyond simple data (subtheme 5.2). Patients described PROMs as helpful prompts for conversation but not substitutes for personal interactions. As one patient noted, *“I sometimes miss that personal touch in the whole process. I find that difficult.”* (P15) This relational dimension is also highlighted by the clinicians: *“It’s nice for the person that’s in contact with me for years that I also contribute to caring for her.”* (P2).

### Theme 6: evaluating and improving PROMs implementation

The final theme captures how participants reflected on the broader value of PROMs beyond individual encounters, going one step further than the scope of the theoretical framework. Clinicians highlighted the potential of PROMs to empower patients (subtheme 6.1), particularly when reflecting on their responses helped patients recognize symptoms or articulate needs more clearly. As one clinician explained, *“The other purpose is to give the patients the opportunity to think about how it’s going*,* how they feel when they’re not in the hospital*,* so they have the time to think about their complaints while filling in the questionnaire.”* (P1) Patients agreed and emphasized that empowerment also depends on how PROMs are designed. They expressed wishes for clearer visualizations, more intuitive interfaces, and results presented in ways that felt meaningful to them. One patient suggested that professionals should pre-test questionnaires to understand the burden and relevance. Together, these comments pointed toward co-design as an important way to more engaging implementation.

Beyond individual empowerment, clinicians emphasized the value of PROMs for organizational learning (subtheme 6.2), particularly through benchmarking within and across institutions. These comparisons help identify where patients stand relative to others and where one institution’s approach may differ from others. Such aggregated insights could, in turn, support the initial stages of PROMs implementation by helping institutions move toward shared standards and understandings of what should be measured, as noted in Theme 1. More broadly, the wishes expressed across themes, including better digital tools (Theme 4) and clearer purpose communication (Theme 2), reflect how evaluation at this final stage feeds back into earlier stages of the implementation process.

## Discussion

This study examined the implementation of PROMs in oncology care in the Netherlands through a communication-centered, sequential lens. While previous research has documented several logistic barriers such as time pressure and inadequate IT infrastructure [15, 30], our findings extend the literature by showing *where* in the implementation process the challenges and opportunities emerge, and how they accumulate across interrelated stages. By empirically mapping challenges onto theoretically informed stages [19], this study helps explain why PROMs sometimes fail to achieve their intended value and highlights how improved communication across stages may enhance patients’ understanding and self-management while supporting healthcare professionals deliver more responsive, person-centered care. Our findings both support and extend the de Ligt et al. framework. Themes 2 through 5 correspond to the framework’s four steps, providing empirical validation for its conceptual structure. Themes 1 and 6 emerged inductively: Theme 1 captures a preparatory organizational stage preceding the framework’s scope, and Theme 6 captures an evaluative stage beyond it.

Examining how this process unfolds, our findings indicate that challenges often originate in early stages, only to become prevalent at later points. In particular, stages that are largely invisible to patients, such as selecting PROMs (theme 1) or evaluating implementation outcomes (theme 6), influence later, more visible stages in which engagement from patients and professionals is expected. For instance, when PROMs selection and task division are fragmented, clinicians have limited opportunities to tailor and introduce PROMs to patients (theme 2), resulting in weaker motivation and engagement (theme 3), and ultimately reduce sense-making and meaningful use of PROMs in care (theme 4 and 5). Conversely, challenges at later stages, such as unmet feedback expectations, also feed back into earlier ones, affecting clinicians’ motivation and organizational decisions, suggesting the process is cyclical rather than strictly linear (see also Fig. 2).

By integrating perspectives from professionals and patients, this study provided additional insights by highlighting experiences that are shared rather than reported from a single group perspective. Across groups, participants recognized the potential of PROMs to raise concerns beyond clinical indicators and to support more person-centered conversations, positioning PROMs not only as a measurement tool [1], but also as instruments facilitating communication between patients and clinicians. At the same time, participants commonly expressed frustration when PROMs were collected but not used, consistent with previous research showing that PROMs are often under-integrated into clinical encounters [31, 32].

Additionally, participants differed in how they interpreted this lack of use. Patients mainly interpreted the absence of feedback as a lack of recognition of their input, which reduced their motivation to engage with PROMs. However, clinicians emphasized that PROMs data are valuable but underused due to systemic barriers, as documented in implementation research [15, 30]. This contradiction highlights the bidirectional, reciprocal potential of PROMs as a communication tool [33]: limited clinical use influences patient perceptions of value, which in turn constrains the completing and therefore application of PROMs in communication and care. More broadly, this echoes the doctor–patient communication literature, which frames communication as a reciprocal process shaped by feedback and shared meaning [34, 35].

Although our primary aim was to identify where challenges occur, the interviews also revealed what worked well in current practices and where opportunities are for improvement. In line with previous research [36, 37], patients described PROMs as moments of self-reflection, and clinicians noted their value in revealing concerns that may otherwise remain unaddressed. Both groups emphasized the emotional and interpersonal aspect of PROMs use, noting that questions should be personally relevant and human interaction is essential for PROMs to feel meaningful. Future research should also explore how patients cope with emotionally challenging moments during PROMs completion, and how clinicians address these emotional aspects when interpreting or discussing PROMs results in clinical encounters. The findings also pointed to the empowering potential of PROMs, as patients suggested concrete improvements such as pre-testing questionnaires and designing more user-friendly interfaces. Taken together, these insights resonate with broader discussions on patient involvement and co-design as ways to enhance the communication quality of PROMs across stages [38].

These insights suggest several practical implications. At the preparatory stage, organizational effort is needed to ensure consistent PROMs selection and clearer division of responsibilities. During the invitation stage, brief personalized explanations or introduction to PROMs can help strengthen patient motivation and clarify expectations [39]. At later stages, co-designing the questionnaire content and interface during the preparatory phase could address issues of usability, burden, and relevance, echoing calls for participatory design in PROMs development [38, 40]. Clinicians would benefit from supportive tools, such as AI-assisted summaries or graphical overviews [19, 41]. Together, these implications highlight that effective PROMs implementation requires coordinated effort across all stages of the communicative process, from organizational preparation to continued evaluation.

A key strength of this study is the use of a communication-centered framework, which allowed challenges to be examined as sequential and interdependent, rather than as isolated barriers. Moreover, the multi-perspective design involving patients, clinicians, and implementation staffs enabled examination of how communicative intentions and experiences diverge across roles and stages. Several limitations should be noted. The study was conducted in the Dutch oncology context, involving professionals from multiple healthcare organizations and patients from six different provinces, providing reasonable diversity but still limiting transferability to other healthcare systems. Differences between hospitals and care pathways may impact participants’ experiences in ways that were not fully revealed in interview data, and future research could examine how organizational context influences specific stages more directly. In addition, interviews capture reported experiences rather than real-time PROMs use. Future studies could combine observational methods with targeted interventions at specific stages, such as redesigned invitations, to examine how strengthening one stage influences the rest of the implementation sequence.

## Conclusion

This study examined how PROMs are implemented and experienced in oncology care using a communication-centered, sequential perspective. We found that challenges and opportunities emerge at specific stages of the implementation process and interacted between stages. By showing what and where challenges occur, this study contributes a process-oriented perspective that extends barrier-focused understanding of PROMs implementation and informs more meaningful, patient-centered care practice.

## Supplementary Information

Below is the link to the electronic supplementary material.

Supplementary Material 1

## Data Availability

Research data generated in this project can be requested at the corresponding author.

## Footnotes

1 The horizontal and vertical dimensions of this framework informed the overall structure of the interview guide. The specific content presented within the cells was not used as predefined themes or codes; interview remained open-ended.

2 Some professionals work across multiple oncology domains; therefore, these categories are not mutually exclusive and do not add up to *n* = 7.
