# Supplementary material for: Implementation of patient-reported outcome measures in oncology practice: a communication-centered qualitative study on patient and healthcare professional perspectives
Source: Qual Life Res. 2026 Aug 22;35(9):267. doi: 10.1007/s11136-026-04378-7 (PMC13499845; doi:10.1007/s11136-026-04378-7)
Supplement: Supplementary file 1 [file 11136_2026_4378_MOESM1_ESM.pdf]

# Interview protocol

## Instructions to interviewers

1. **General Topics**: The guide is organized into 4 main topics (found in the first column): *Setting the Stage*, *Current Practice*, *Major Bottlenecks*, and *Towards Solutions*. Each topic is designed to explore different aspects of PROMs implementation.
2. **Specific Questions**: Under each general topic, you will find a set of specific questions (listed in the second column). These questions are intended to elicit detailed responses about current practices, challenges, and potential improvements.
3. **Prompts**: To encourage deeper discussion or to clarify responses, prompts for each question are provided (located in the third column). Use these prompts as needed to facilitate a more comprehensive dialogue.
4. **Framework for the process**: The figure below outlines an overall framework for the collection and communication processes around PROMs in a clinical setting. The *vertical dimension* represents various potential steps involved in the process, while the *horizontal dimension* shows people/organizations involved at each step, as well as their actions. This framework is intended to provide context for asking questions in the *Current Practice* and *Major Bottlenecks* sections, guiding you to explore how stakeholders interact across the process. Use the framework for inspiration when formulating questions, but you are not limited to the elements included here. Please note that this framework is for your reference only and should not be shown to the participants. Please try to gather information about as many elements as possible in the framework, especially "in what form", which brings valuable insights for later analysis.

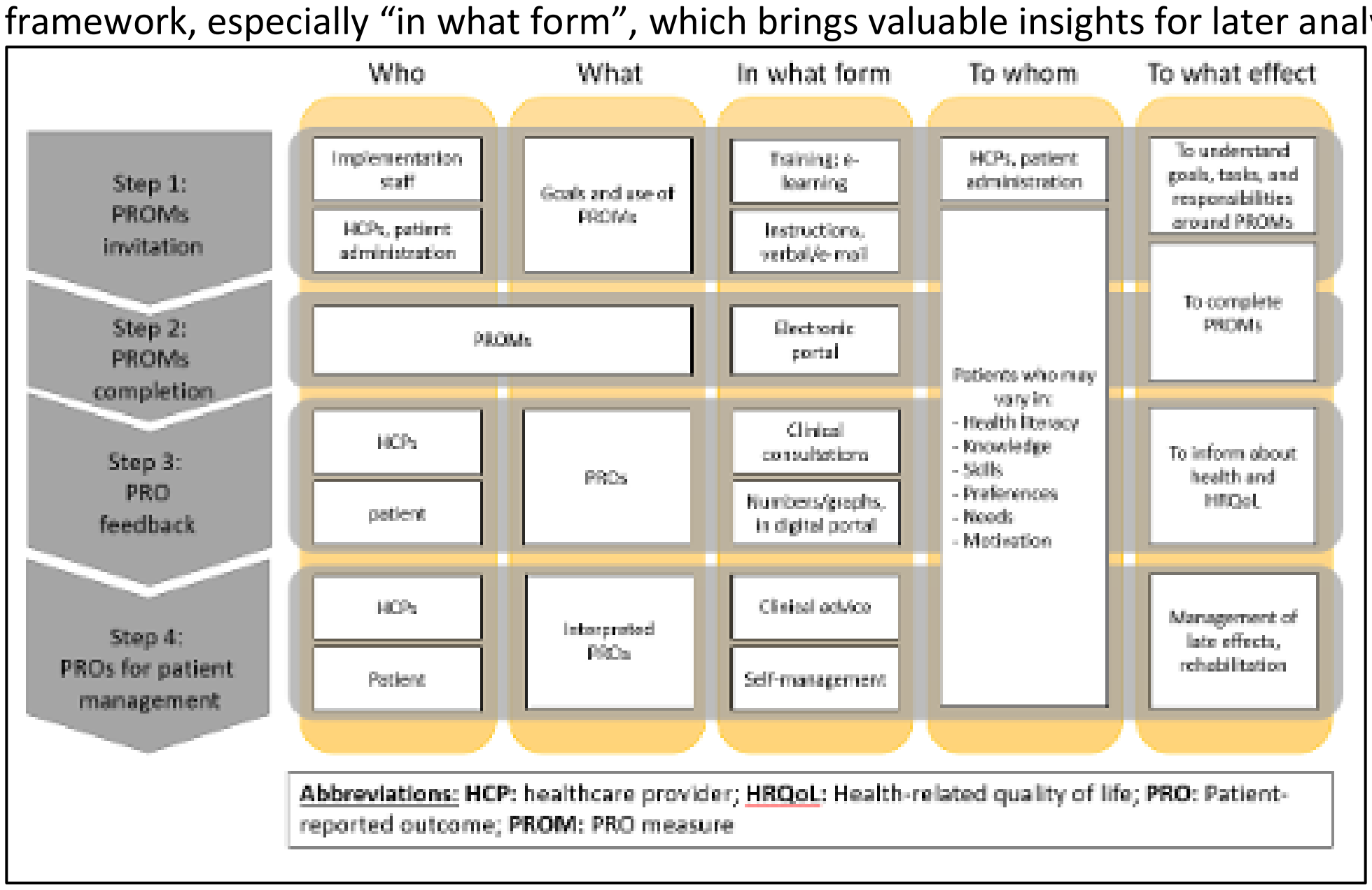

**Interview guide**

| Topics | Questions | Prompts |
|---|---|---|
| **Introduction** | ***Please avoid asking questions regarding personal information, focus on categories and information on aggregated levels***<br><br>Q1: Could you please tell me about yourself and your work, specifically,<br>- Country/region where you work/live<br>- The type of organization you are affiliated with (hospital, company, government institution, etc.)<br>- Your role within your organization<br>- Your areas of expertise and years of experience<br>- [If applicable, as a patient] Your disease area | |
| **Setting the stage** | ***INTRO:***<br>The next few questions are going to help us set the stage, understand the context of how you encounter/need/make use of PROMs. | |
| | ****Role of PROMs ****<br><br>***IF non-patient:***<br>Q2: Can you describe the role of PROMs in your organization? Specifically, for what purposes do you collect them?<br><br>Q3: How would you describe the role of PROMs to someone new to your organization?<br><br>***IF patient:***<br>Q2: When you first heard about PROMs, how were they explained to you? Did you understand their purpose right away?<br><br>Q3: How are PROMs used in your healthcare experience? What purpose do they serve in your interactions with healthcare providers? | **Non-patient:**<br>- What is the primary benefit of collecting PROMs? What do you hope to **achieve** by using them?<br>- Do you believe these measures are well-received by staff and patients?<br>- What key purposes or goals would you highlight when introducing these measures to someone new?<br><br>**Patient**:<br>- Why do YOU think your healthcare provider asks for your input through these measures? |
| | ****Perceived importance/impact of PROMs**** | **Non-patient:**<br>- How do these influence the outcomes or quality of care you provide? |

| | | |
|---|---|---|
| | Q4: How important do you think it is for YOU to collect them? Why? | - In what ways do these align with your organization's goals or objectives?<br>- Examples of how these have impacted your work (workflow, decision-making…)?<br><br>**Patient:**<br>- Examples of how these have impacted your overall healthcare experience?<br>- How do they impact your interaction with your healthcare provider? |
| **Current practice** | ***INTRO:***<br>To take a step further, the next set of questions will focus on how you currently work with PROMs. We'll be exploring the various steps involved in the process, the people or resources you collaborate with, and any factors that play a role in your experience. This will help us better understand the current practices and any challenges you may face in working with PROMs. | |
| | Q5: Could you describe what steps are involved in your work/experience related to PROMs?<br><br>Q6: how is the data you collected currently being used? | ***Use the framework for prompts***<br>- Why are some steps not involved/necessary?<br>- Which steps receive the most attention/resource, and why? |
| | Q7: For the steps you described, could you tell a bit more about them? For example, who is responsible for step x, and how are they carrying out the task? (move to prompts) | ***Use the framework for prompts***<br>- Who is responsible for each step?<br>- How do they carry out the tasks (in what form)?<br>- What is the purpose for each step? |
| **Major bottlenecks** | Q8: What are some major bottlenecks you experience in this process? | ***Use the framework for prompts***<br>- In which steps (the vertical dimension)?<br>- With which actors or aspects (the horizontal dimension)?<br>- Source of the challenges? (resource, technology, personnel…) |
| **Towards solutions** | ***INTRO:***<br>To potentially address these challenges, we're working on a project focusing on using patient-generated health data with m-health and e-health tools to tackle some of the key gaps in current healthcare systems. Specifically, it's designed to improve three areas. First, the project aims to better incorporate patient outcome into treatment | |

| | | |
|---|---|---|
| | decisions, so care is more tailored to what each person actually needs. Another goal is to use patient feedback to shape how new medical devices are designed, making sure they fit better with what patients expect and need in their daily lives. Finally, the project wants to help get patient-focused and cost-effective treatments to the market faster, so people have better access to new and effective care solutions.<br><br>We're working to build a platform that integrates all these elements and uses real-world data to drive smarter, patient-centered healthcare decisions.<br><br>In the next few questions, we'll talk about how you think this project might address the challenges we've discussed and what solutions or ideas could help move things forward. | |
| | Q9: In the context of the project's goal to use m-health and e-health technologies, what specific functionalities or features would you like to see that could better capture and utilize PROMs? | ***Use the framework for prompts***<br>- In which steps (the vertical dimension) do you see the potential improvement?<br>- With which actors or aspects (the horizontal dimension)? Are there additional elements needed? |
| | Q10: Consider your profession and your day-to-day life, and think about the project's goal to build a platform to better integrate the collection, use, and feedback of the PROMs data, what do you perceive as potential challenges or barriers to implementing such a platform? How can these challenges be addressed to ensure successful adoption and integration into your existing processes? | |
| **Conclusion** | Is there anything else you would like to share? | |
